# Supplementary material for: Pulmonary haemorrhage as a frequent cause of death among patients with severe complicated Leptospirosis in Southern Sri Lanka
Source: PLoS Negl Trop Dis. 2023 Oct 16;17(10):e0011352. doi: 10.1371/journal.pntd.0011352 (PMC10602373; doi:10.1371/journal.pntd.0011352)

**Supplementary Figure 1**: Serial chest radiographs showing gradual resolution of pulmonary shadows in a patient who underwent ECMO. (Chest radiographs were taken from different machines) Day 1 & 2 were taken on presentation before initiating ECMO, others were taken during and before weaning from ECMO.


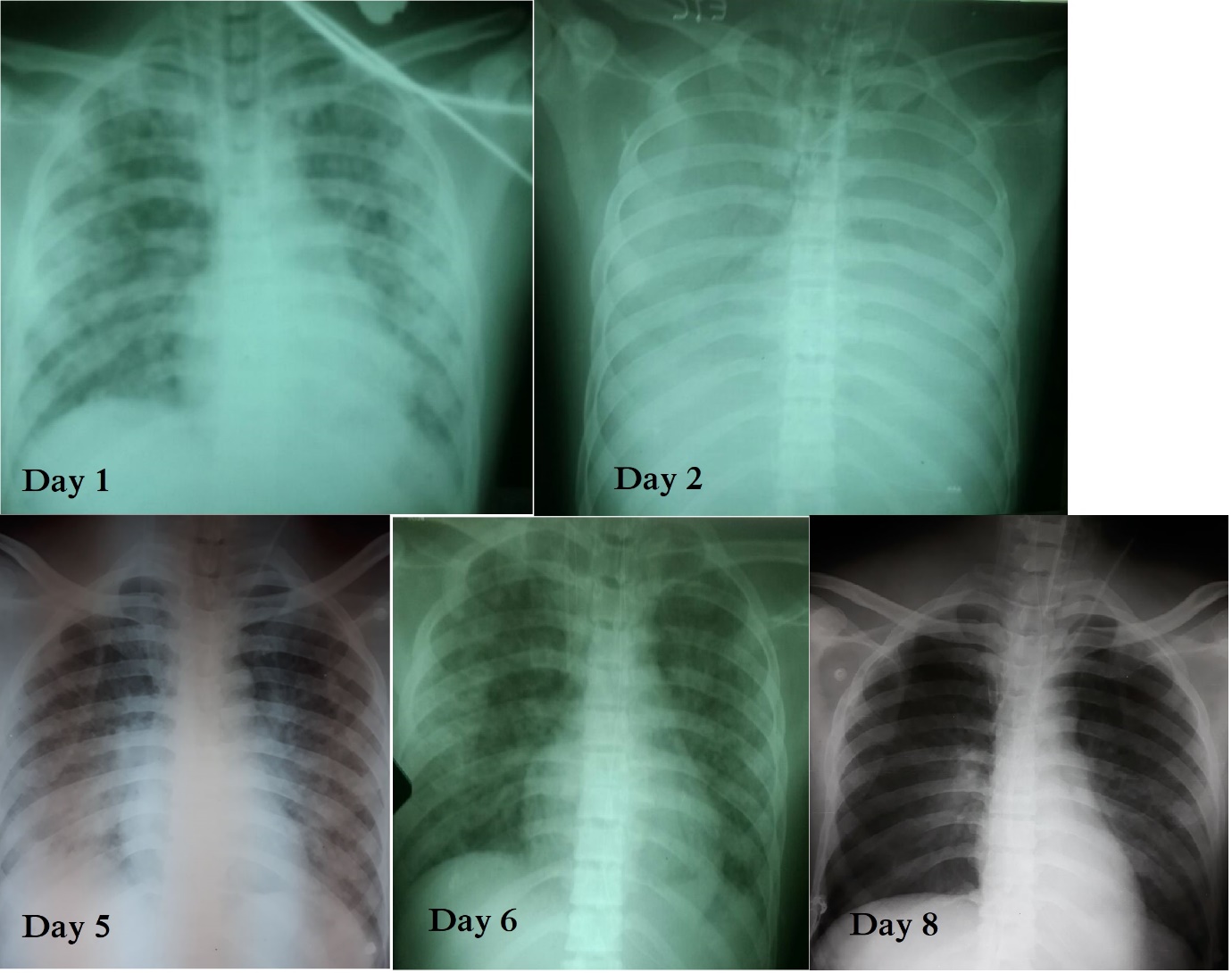

Supplement: S1 Fig — (DOCX) [file pntd.0011352.s001.docx]
