# Supplementary material for: Pulmonary haemorrhage as a frequent cause of death among patients with severe complicated Leptospirosis in Southern Sri Lanka
Source: PLoS Negl Trop Dis. 2023 Oct 16;17(10):e0011352. doi: 10.1371/journal.pntd.0011352 (PMC10602373; doi:10.1371/journal.pntd.0011352)
Supplement: S1 Table — (DOCX) [file pntd.0011352.s002.docx]

**Supplementary Table 1:** Leptospirosis testing data for Rapid IgM, PCR and Microscopic Agglutination Test (MAT).

**Diagnosis of Leptospirosis in patients clinically suspected of leptospirosis**

|  | **Number of patients** |
| --- | --- |
| Leptospirosis IgM positive | 15 |
| PCR positive | 22 |
| MAT positive | 83 |
| PCR positive MAT negative | 5 |

A single Microscopic Agglutination Test (MAT) titre ≥1:400 and/or by detection of Leptospira DNA by PCR and/or by the presence of IgM antibodies.

| **Number** | **IgM** | **PCR** | **MAT** |
| --- | --- | --- | --- |
| 1 | 1 | - | 1 |
| 2 | - | - | 1 |
| 3 | - | - | 1 |
| 4 | - | 1 | 1 |
| 5 | 1 | - | 1 |
| 6 | - | 1 | 2 |
| 7 | - | - | 1 |
| 8 | - | 2 | 1 |
| 9 | - | - | 1 |
| 10 | 1 | - | 1 |
| 11 | - | - | 1 |
| 12 | - | - | 1 |
| 13 | - | 1 | 1 |
| 14 | - | 1 | 1 |
| 15 | - | 1 | 1 |
| 16 | 1 | - | 1 |
| 17 | - | 1 | 1 |
| 18 | - | - | 1 |
| 19 | 1 | 1 | 1 |
| 20 | - | - | 1 |
| 21 | - | - | 1 |
| 22 | - | - | 1 |
| 23 | - | 1 | 1 |
| 24 | 1 | - | 1 |
| 25 | - | - | 1 |
| 26 | - | - | 1 |
| 27 | - | - | 1 |
| 28 | - | - | 1 |
| 29 | 1 | 2 | 1 |
| 30 | - | - | 1 |
| 31 | - | - | 1 |
| 32 | - | - | 1 |
| 33 | - | 1 | 1 |
| 34 | - | - | 1 |
| 35 | 1 | 1 | 1 |
| 36 | - | 2 | 1 |
| 37 | 1 | - | 1 |
| 38 | 1 | - | 1 |
| 39 | - | 1 | 2 |
| 40 | - | - | 1 |
| 41 | - | - | 1 |
| 42 | - | 2 | 1 |
| 43 | - | 1 | 1 |
| 44 | 1 | - | 1 |
| 45 | - | - | 1 |
| 46 | - | - | 1 |
| 47 | - | - | 1 |
| 48 | - | 1 | 1 |
| 49 | - | - | 1 |
| 50 | - | - | 1 |
| 51 | - | - | 1 |
| 52 | - | - | 1 |
| 53 | 1 | - | 1 |
| 54 | - | - | 1 |
| 55 | - | - | 1 |
| 56 | - | 1 | 2 |
| 57 | - | - | 1 |
| 58 | - | - | 1 |
| 59 | - | - | 1 |
| 60 | - | - | 1 |
| 61 | - | 1 | 1 |
| 62 | - | - | 1 |
| 63 | - | 1 | 1 |
| 64 | - | - | 1 |
| 65 | - | - | 1 |
| 66 | - | - | 1 |
| 67 | - | 1 | 2 |
| 68 | 1 | - | 1 |
| 69 | - | - | 1 |
| 70 | - | - | 1 |
| 71 | - | - | 1 |
| 72 | - | - | 1 |
| 73 | - | 1 | 1 |
| 74 | - | - | 1 |
| 75 | - | - | 1 |
| 76 | - | - | 1 |
| 77 | 1 | - | 1 |
| 78 | - | 1 | 1 |
| 79 | - | 1 | 2 |
| 80 | - | - | 1 |
| 81 | - | - | 1 |
| 82 | - | 1 | 1 |
| 83 | - | - | 1 |
| 84 | - | - | 1 |
| 85 | - | - | 1 |
| 86 | - | - | 1 |
| 87 | - | 1 | 1 |
| 88 | 1 | 2 | 1 |
