# Supplementary material for: Pulmonary haemorrhage as a frequent cause of death among patients with severe complicated Leptospirosis in Southern Sri Lanka
Source: PLoS Negl Trop Dis. 2023 Oct 16;17(10):e0011352. doi: 10.1371/journal.pntd.0011352 (PMC10602373; doi:10.1371/journal.pntd.0011352)
Supplement: S2 Table — (DOCX) [file pntd.0011352.s003.docx]

**Supplementary Table 2: Summary table depicting data of patients who underwent ECMO having pulmonary haemorrhage due to Leptospirosis**

| **No.** | **Age,**  **Gender** | **Day of PH** | **Total ECMO**  **Hours** | **P/F**  **Ratio (Before ECMO)** | **Murray**  **Score (Before ECMO)** | **Organ**  **Involvement** | **Serum**  **Creatinine (Before ECMO)** | **Plasmapheresis**  **(Cycles)** | **HD** | **CRRT** | **Intub.**  **Days** | **ICU**  **Days** | **Total**  **Hospital days** | **ECMO related**  **Complications** | **Outcome** |
| --- | --- | --- | --- | --- | --- | --- | --- | --- | --- | --- | --- | --- | --- | --- | --- |
| 1 | 34,M | D6 | 148 | 78 | 3.5 | PH,AKI | 522 | Yes (1) | Yes | No | 13 | 18 | 21 | VAP (Klebisiella) | Alive |
| 2 | 18,M | D5 | 202 | 157 | 3.0 | PH,AKI | 369 | Yes (2) | Yes | Yes | 15 | 15 | 28 | None | Death |
| 3 | 55,M | D7 | 88 | 132 | 3.25 | PH,AKI | 591 | Yes (3) | Yes | No | 24 | 24 | 40 | VAP | Alive |
| 4 | 48,M | D12 | 143 | 93 | 3.25 | PH,HI,AKI | 195 | Yes (4) | No | No | 10 | 17 | 22 | None | Alive |
| 5 | 54,M | D8 | 97 | 86 | 4 | PH,HI,AKI | 848 | No | No | Yes | 8 | 9 | 10 | Bleeding due to DIC | Death |
| 6 | 36,M | D5 | 114 | 81 | 3.5 | PH,AKI | 169 | Yes (7) | No | No | 5 | 8 | 11 | Bleeding due to DIC | Alive |

*PH – Pulmonary Hemorrhage, SS – Haemodynamic instability with inotrope requirement, AKI – Acute Kidney Injury, HD – Hemodialysis, CRRT – Continuous renal replacement therapy, VAP – Ventilator associated pneumonia
